# From mice to men

**DOI:** 10.7554/eLife.67895

**Published:** 2021-04-01

**Authors:** J Christian Althaus, Michael A Sutton

**Affiliations:** 1 Department of Molecular and Integrative Physiology, University of MichiganAnn ArborUnited States; 2 Michigan Neuroscience Institute, University of MichiganAnn ArborUnited States

**Keywords:** synaptopodin, synaptic plasticity, human cortex, vitamin A, retinoic acid, Human, Mouse

## Abstract

All-trans retinoic acid induces functional and structural plasticity of synapses in human cortical circuits through the engagement of the spine apparatus.

**Related research article** Lenz M, Kruse P, Eichler A, Straehle J, Beck J, Deller T, Vlachos A. 2021. All-trans retinoic acid induces synaptic plasticity in human cortical neurons. *eLife* **10**:e63026. doi: 10.7554/eLife.63026

Over the last decade, the synapses that connect neurons have emerged as important therapeutic targets in a host of neurological disorders ranging from autism to Alzheimer’s disease. Synaptic signaling can either excite or inhibit the postsynaptic neuron, and the vast majority of excitatory synapses in the mammalian brain rely on structures called dendritic spines (Figure 1). The 'head' of each dendritic spine contains receptors for the chemical neurotransmitter glutamate, which is released by the presynaptic neuron. Neurons can contain different types of glutamate receptors, but the AMPA-type glutamate receptor (AMPAR) is responsible for the majority of fast synaptic transmission and also controls the strength of the synapse.

**Figure 1.**
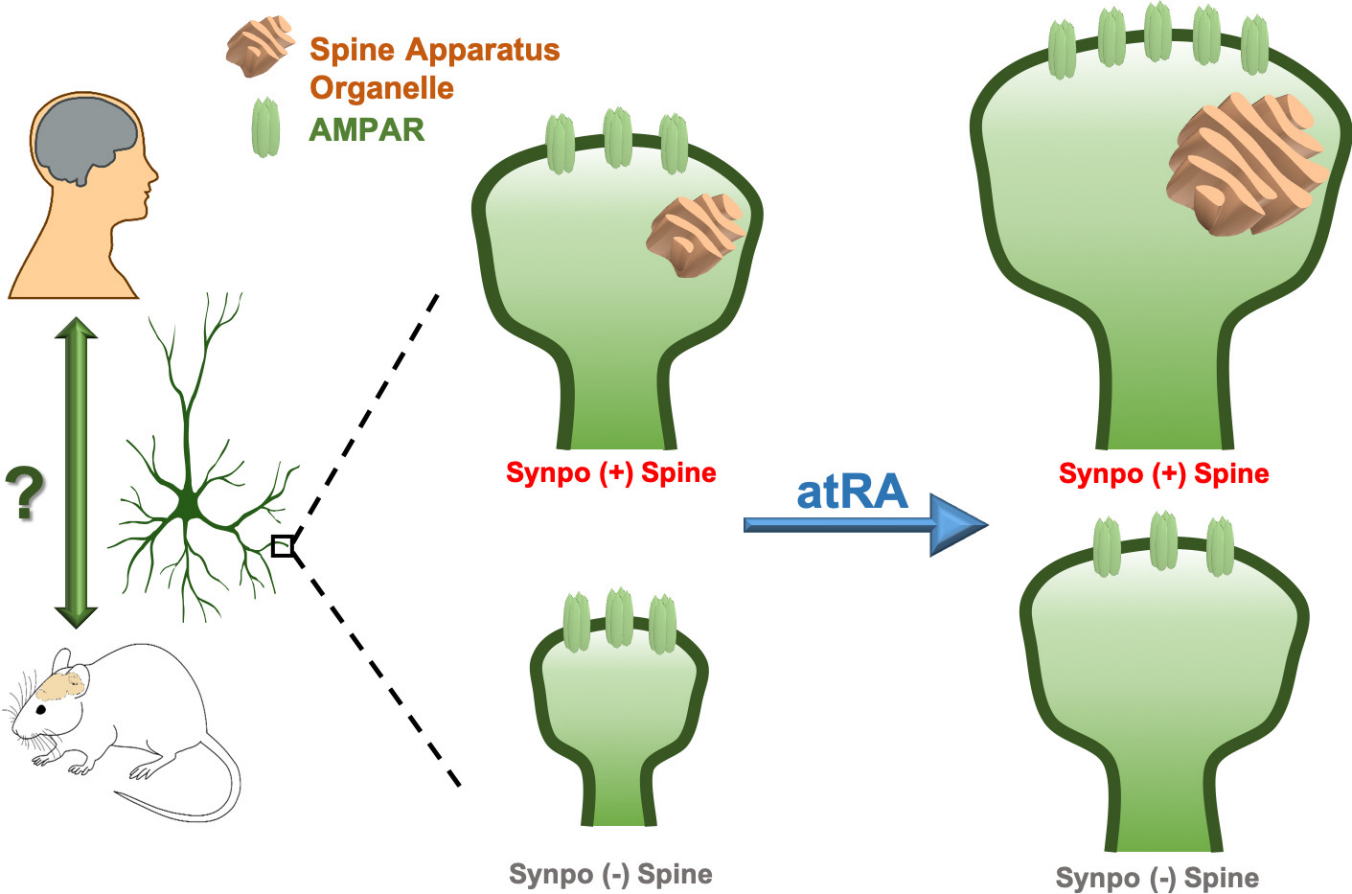
Remodeling human cortical synapses with all-trans retinoic acid. Left: the synapse modulating effects of all-trans retinoic acid (atRA) first reported in rodent neurons are preserved in human cortical neurons in intact cortical circuits. Right: all-trans retinoic acid increases the strength (measured as the number of AMPA-type glutamate receptors; AMPARs, green) and size of excitatory synapses in layer 2/3 pyramidal neurons in human cortical slices. All-trans retinoic acid also increases the size of the spine apparatus, a synaptic organelle found in dendritic spines and previously linked to synapse remodeling (orange). To test whether the spine apparatus is important for the effects of all-trans retinoic acid on synapses, Lenz et al. compared wild-type (Synpo +, top), and synaptopodin-deficient (Synpo -, bottom) mice, which lack the spine apparatus. Synapses lacking the spine apparatus were smaller and failed to increase in strength after applying all-trans retinoic acid. However, applying the molecule still enlarged the spines without a spine apparatus, demonstrating that this organelle has a specific role in regulating changes in synaptic strength induced by all-trans retinoic acid.

Many forms of synaptic plasticity – the process that allows specific synapses to become stronger or weaker over time – rely on the addition and removal of AMPARs (Diering and Huganir, 2018). These changes are often accompanied by an increase or decrease in the size of the dendritic spine head (Matsuzaki et al., 2004). Changes in synapse number or strength are a pathological hallmark of several diseases, including neurodevelopmental disorders and Alzheimer’s disease (Forrest et al., 2018), which means that molecules that can consistently modify synapses are attractive as potential therapeutics. Among the most promising of these is a derivative of vitamin A called all-trans retinoic acid, which can potently increase synaptic strength in cultured rodent neurons (Aoto et al., 2008). This molecule appears to function as part of a homeostatic pathway that engages the protein translation machinery in dendritic spines to increase the strength of synapses when synaptic input drops (Jakawich et al., 2010; Wang et al., 2011).

Since all-trans retinoic acid is a potent synaptic regulator with a well-defined mechanism of action, it offers tremendous promise to guide therapeutic development for disorders characterized by synaptic dysfunction. But the critical question is whether these effects and mechanisms are readily translatable to the human brain. Now, in eLife, Andreas Vlachos of the University of Freiburg and colleagues – including Maximilian Lenz as first author – report striking parallels between human and rodent neurons in the synaptic effects of all-trans retinoic acid (Lenz et al., 2021).

The researchers prepared slices from surgically resected brain tissue from patients undergoing neurosurgery to ask whether all-trans retinoic acid has the same effects on human pyramidal neurons from layer 2/3 of the cortex as it has on rodent neurons. They found that the molecule enhanced synaptic currents, without altering many other features of neuronal excitability. Along with these changes, Lenz et al. found that all-trans retinoic acid drove enlargement of dendritic spine heads, but the overall density of dendritic spines did not change: this suggests that all-trans retinoic acid drives AMPAR accumulation and structural plasticity at pre-existing synaptic sites. Finally, Lenz et al. demonstrated that the changes in synaptic strength induced by all-trans retinoic acid in human neurons depended on mRNA translation but not on transcription, a mechanistic signature first seen in rodent neurons.

Next, Lenz et al. – who are based at the University of Freiburg and Goethe-University Frankfurt – explored the relationship between synaptic modulation by all-trans retinoic acid and the spine apparatus, an organelle that is present in a subset of dendritic spines and whose function has remained enigmatic (Jedlicka and Deller, 2017). They found that all-trans retinoic acid enlarged the spine apparatus and, strikingly, that the cross-sectional area of the spine apparatus varied with the size of the dendritic spine itself. This suggests that the spine apparatus might have a key role in the modulation of synaptic strength by all-trans retinoic acid.

To test this hypothesis, Lenz et al. examined the effects of all-trans retinoic acid in synaptopodin knockout mice, which lack the spine apparatus. They found that all-trans retinoic acid did not enhance synaptic currents in cortical pyramidal neurons in the knockout mice; however, when synaptopodin was reintroduced, all-trans retinoic acid was able to increase synaptic currents. Curiously, losing the spine apparatus did not prevent all-trans retinoic acid from enlarging dendritic spines, even though spine size was reduced in the synaptopodin knockout mice.

These results suggest that the spine apparatus helps regulate synaptic architecture, but that all-trans retinoic acid can induce structural remodeling of synapses independently of this organelle. This finding was particularly intriguing because the enlargement of spines and the enhancement of synaptic function often go hand-in-hand. These results underscore the fact that while morphological and functional changes are highly coordinated at cortical synapses, they likely rely on distinct mechanistic pathways.

These results provide a clear answer as to whether the effects of all-trans retinoic acid in rodents can be translated to humans. The molecule is a potent regulator of excitatory synapses in human cortical neurons and uses a mechanism for synaptic regulation that appears largely conserved from rodents to humans. The work of Lenz et al. also raises some important questions about the role of the spine apparatus in the regulation of synapses by all-trans retinoic acid in particular, and by other modulators more generally.

It is tempting to speculate that the spine apparatus may be part of a satellite secretory pathway that delivers locally-translated membrane proteins, such as AMPARs, in dendrites. However, future studies are needed to address the specific role of the spine apparatus relative to other secretory mechanisms that might also operate in dendrites (Pierce et al., 2001; Mikhaylova et al., 2016).
